# Three-dimensional printing designed customized plate in the treatment of coronal fracture of distal humerus in teenager: A case report

**DOI:** 10.1097/MD.0000000000032507

**Published:** 2023-01-13

**Authors:** Changpeng Cao, Haiyang Xing, Faxin Cao, Zhipeng Du, Gang Wang, Xiyao Wang

**Affiliations:** a China-Japan Union Hospital of Jilin University, Changchun City, Jilin Province, China.

**Keywords:** 3-dimensional printing designed, children, coronal shear fracture of distal humerus, customized plate, shear force, teenager

## Abstract

Because of the complicated anatomy and considerable change in size and morphology with age in teenagers, the appropriate internal fixator of coronal shear fracture of distal humerus is difficult to choose, and therefore, the fixation of this kind of fracture is difficult and controversial. Furthermore, distal humeral fractures in teenagers often involve the epiphysis, the rigid fixation of fracture and the simultaneous minimally invasive and protection of the epiphysis are contradictory. Coronal shear fractures of the distal humerus in teenagers are great challenge for orthopedic surgeons. Three-dimensional (3D) printing designed customized plate in the treatment of coronal fracture of distal humerus in teenager is a potential satisfactory choice in the treatment of the complex fractures.

**Patient concerns:**

A teenager suffered from an elbow joint injury due to a fall while running, resulting in pain, swelling and limited movement of the elbow joint. The epiphyseal has not closed in this patient, conventional surgical procedures have great traumatic and invasive, and to some extent affect bone growth in children.

**Diagnoses:**

Coronal shear fracture of right distal humerus according to computed tomography scan.

**Interventions:**

We used 3D printing technology to design an internal fixation device for this patient, which was to treat the distal humeral coronal shear fracture in a teenager via an anterior approach to the elbow joint, and finally the child was instructed to perform immediate postoperative functional exercises and rehabilitation.

**Outcomes:**

Radiographic reexamination performed 1 day and 2 month after the operation showed that the internal fixation was in good position, no fracture displacement. the patient was instructed to perform active flexion and extension internal and external rotation of the right elbow 6 weeks postoperatively. The Mayo elbow function score was excellent 5 months postoperatively. The range of motion of the elbow was (15°–130°)

**Lessons:**

The treatment of coronal shear fractures of the distal humerus in teenager is controversial at present. This report 3D printing technology designed customized plate in treatment of such fractures showed satisfactory results, which provides a feasible method for the treatment of fractures without suitable internal fixation devices in the future.

## 1. Introduction

Distal humeral fractures account for approximately 30% of elbow fractures, and the coronal shear fractures of the distal humerus account for only about 1% of all elbow fractures and 3% to 6% of distal humeral fractures,^[1–3]^ which are rare and difficult to treat. The anatomical morphology of the distal humerus changes considerably with age during growth and development in children.^[4,5]^

Arbeitsgemeinschaftfür osteosynthesefragen classification of distal humeral fractures can be divided into: Type A: complete extraarticular fracture; Type B: partial intra-articular fracture; Type C: complete intra – articular fracture.^[1,2]^ Type B partial intra-articular fractures include type B1: Partial intra-articular fracture involving the lateral condyle; Type B2: partial intra-articular fracture involving the medial condyle; And type B3, the fractures are the most difficult fractures in reduction and fixation, involving a coronal shear fracture of the capitellum and/or trochlea humeri.

The coronal shear fracture of distal humerus fracture is mainly caused by low-energy injuries such as falls. There are 2 injury mechanisms at present. The first is an indirect impact along the radius in full extension, which results in a vertical shear stress on the distal humerus caused by the radial head. Another mechanism is that the shear force generated during reduction of the radial head after posterolateral dislocation or subluxation of the elbow acts on the capitellum, the shear force resulting in fracture.^[2,3]^

Appropriate postoperative exercise can be done as early as possible. The restoration of anatomical reduction and rigid fixation of intra-articular fracture of the elbow joint is indispensable for postoperative exercise.^[6]^ At present, interference of epiphysis of conventional surgery is not controllable and there is no suitable anatomical internal fixation device for the distal humerus in teenagers before epiphysis closure. With the development of three-dimensional (3D) printing design technology, more and more orthopedic patients benefit from it. In this case report, a 3D printing designed customized internal fixation device was applied to withstand the high shearing force which was difficult to fix with suitable morphology and size for the particular individual. This customized internal fixation is mechanically stable, preventing internal fixation failure and fracture displacement, and allows for early functional exercise and restoration of elbow function.^[7]^

## 2. Case presentation

**Figure 1.**
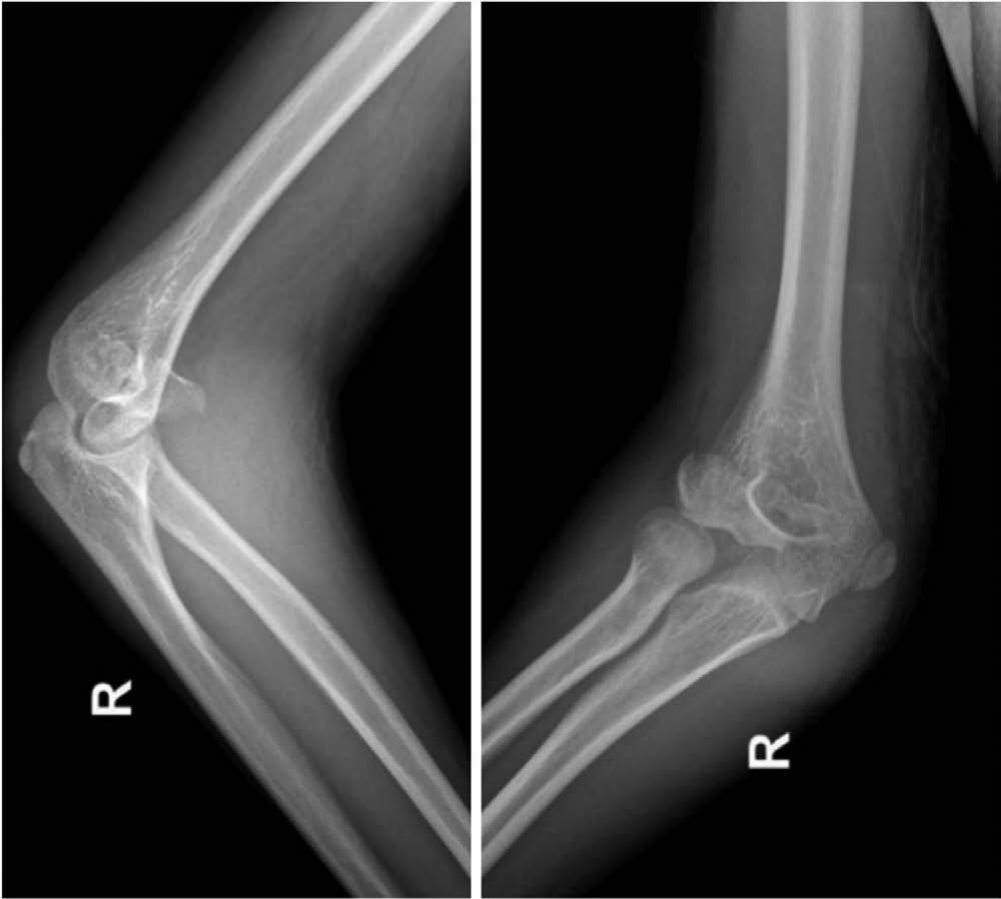
X-ray of the right elbow.

A 14-years-old male patient suffered from a fall while running and presented with pain and swelling of the right elbow joint combined with restricted movement of the elbow joint for 1 day. The ulnar deviation test (+). Combined with physical examination and X-rays (Fig. 1) the patient was diagnosed as a fracture of the right distal humerus, injury of the radial collateral ligament of the right elbow, and avulsion fracture of the right olecranon. There were no open wounds and no neurovascular injury.

**Figure 2.**
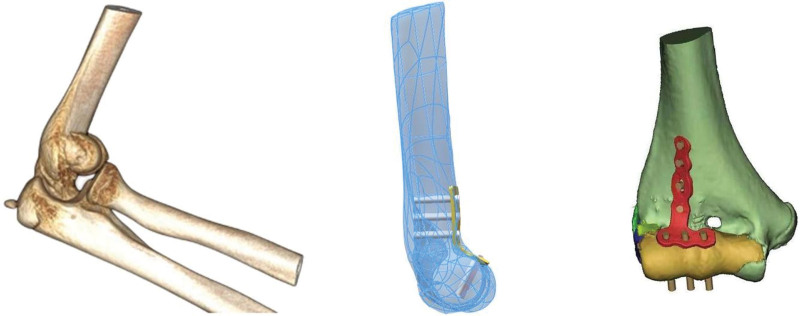
3D reconstruction model and internal fixation method.

We reconstructed the 3D model of the fracture based on the DICOM data from computed tomography (CT) scan of fracture limb. We also reconstructed the normal unfractured model of distal humerus based on the inversion mirror image of CT scan of the contralateral healthy humerus. The internal fixation devices and fixation strategies were designed according to the 3D printed model of both fracture and the fracture after anatomical reduction.(Fig. 2)

### 2.1. Treatment process

A longitudinal incision about 7 cm in length was made slightly lateral to the anterior median of the humerus, and the basilic veins was carefully isolated and protected. The biceps brachialis and brachialis muscles were retracted ulnaris, and the brachioradialis muscle was split longitudinally to expose the joint capsule. Brachial artery and median nerve were under protection.

**Figure 3.**
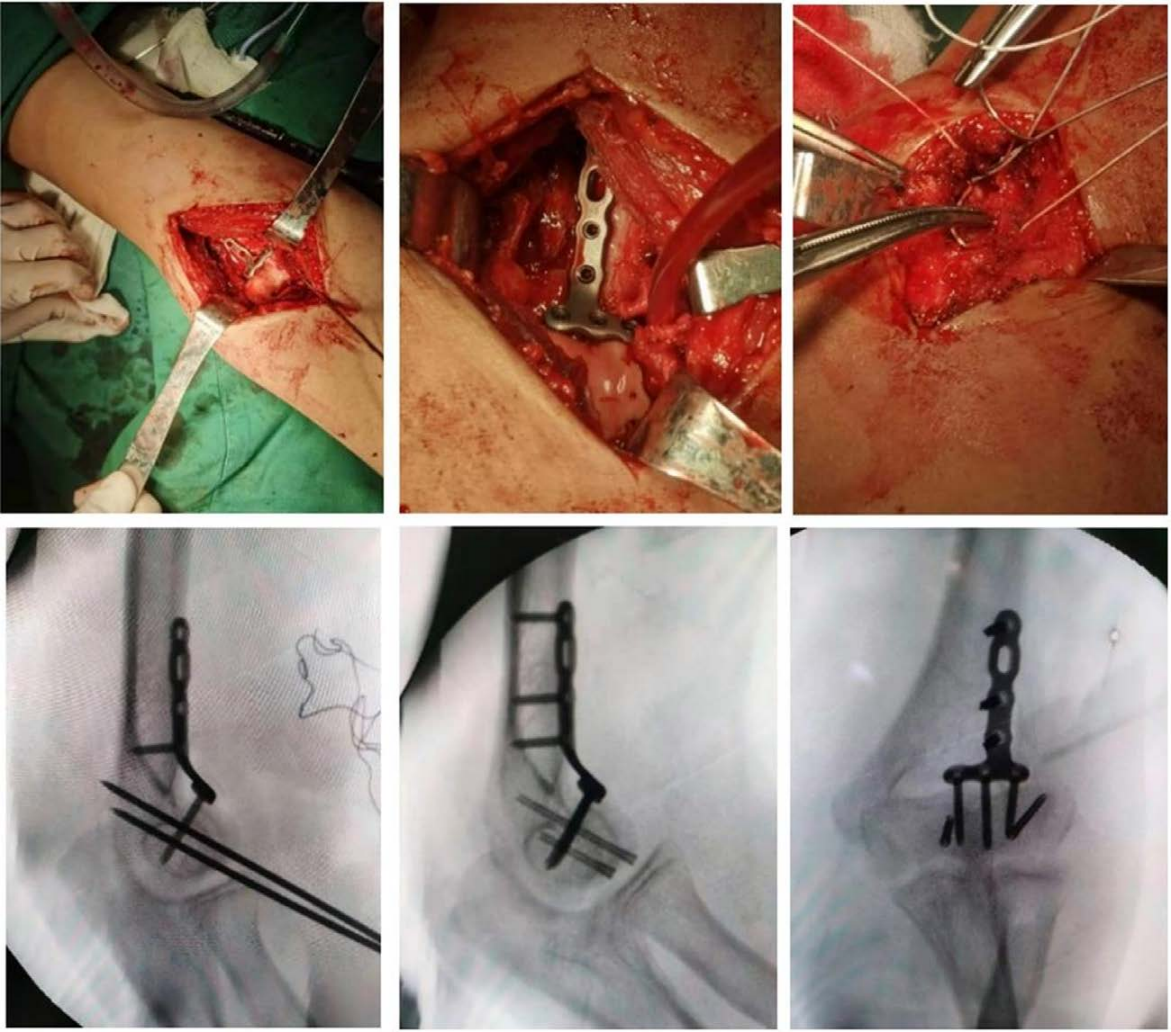
Intraoperative images.

A coronal shearing fracture block of the distal humerus was seen with a proximal displacement of approximately 1 cm. After anatomical reduction of the fracture fragment, Two Kirschner wires were inserted perpendicular to the fracture line in the anatomical axis of the fracture block for temporary fixation, and in turn, the Kirschner wires were replaced by 2 cannulated screws. The 3D printing designed anti-slide plate was attached to the anterolateral distal humerus and screwed into the locking screw for fixation. The radial collateral ligament was repaired to restore the radial stability of the elbow joint, The extensor carpi radialis longus brevis was bluntly separated at the lateral elbow, the broken point was at the origin end of the lateral collateral ligament on lateral condyle of the humerus. The 5 mm diameter bone anchor was inserted into the bone cortex at the footprint of the origin of lateral collateral ligament, and the ligament was repaired with 2 strands of ultra-braid thread with Kessler suture. Varus test negative showed the restoration of stability of the elbow joint. (Fig. 3: Intraoperative images)

After fixation, the elbow joint was stable within the range of flexion and extension from 0° to 140 °, and the humeral ulnar joint was well aligned without laxity. Re-fluoroscopy showed anatomical reduction and stable fixation of the fracture. The patient’s intra-operative bleeding was about 100 mL. After operation, the elbow joint was fixed in 90° flexion., Early functional exercise of finger, wrist and shoulder joints were permitted. The surgical incision healed without any complications, and the patient was discharged 5 days after operation.

**Figure 4.**
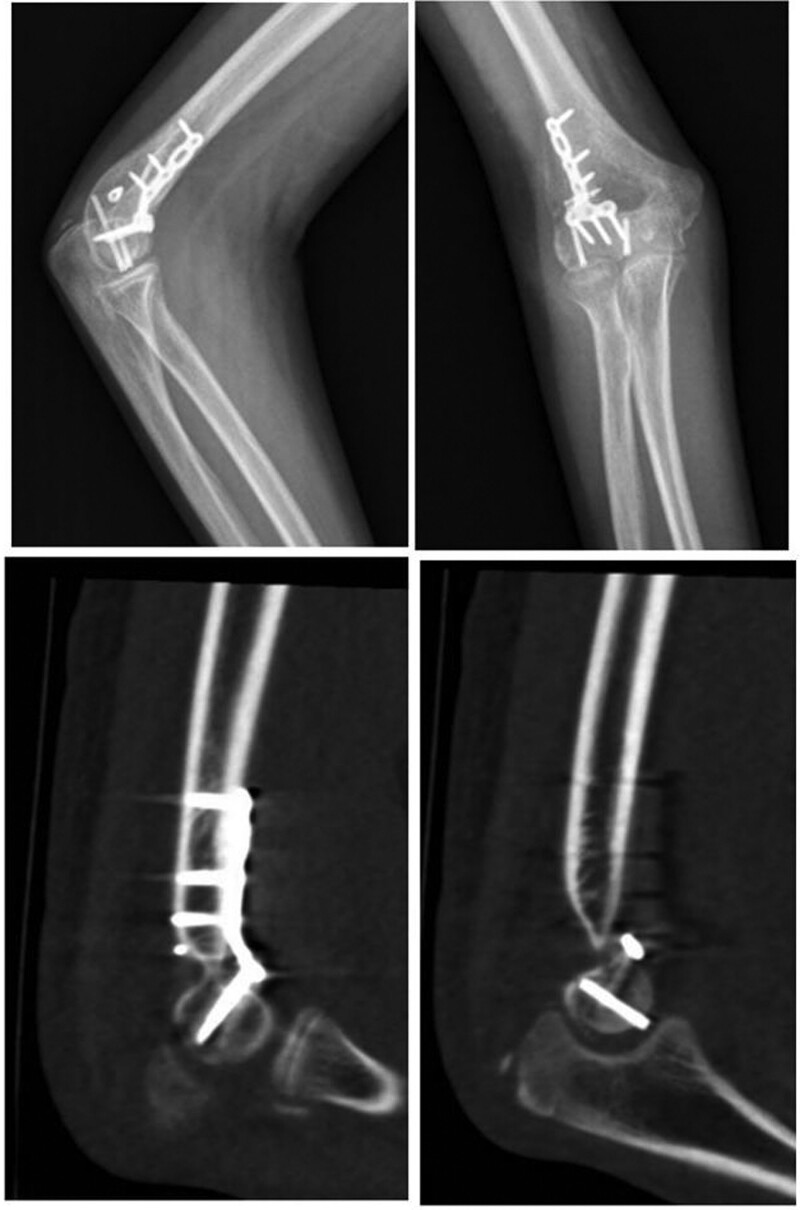
Postoperative reexamination.

Postoperative X-ray and CT of elbow: The fracture was anatomically reduced and the internal fixation device was reliable. (Fig. 4)

**Figure 5.**
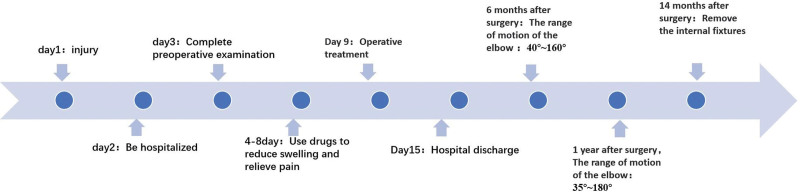
The course of treatment.

The elbow was stabilized in a cast for 2 weeks. After removal of the cast was, the patient was instructed to do active and passive flexion and extension exercises of the elbow joint. Postoperative x-ray examination at 1 day and 1 month showed satisfactory fracture reduction, good position of the internal fixator, and stable internal fixation without fracture displacement. Six weeks after surgery, the patient was instructed to perform active flexion and extension, internal and external rotation of the elbow. Finally the internal fixation device was removed at 14 months after surgery, and we found that the Post-operative scar was formation, we need help children to remove that. (Fig. 5: The course of treatment)

The Mayo elbow function score at 5 months and 1 year after surgery was 40° to 160°/35° to 180°: excellent.(Table 1)

**Table 1:** The Mayo elbow function score.

Pain (45 points)	No pain	Slight	Moderate	Severe
	45	30	15	0
	√			
Motion (20 points)	>100°	50–100°	<50°	
	20	15	5	
	√			
Stability (10 points)	Stabilize	Moderate	Instability	
	10	0	0	
	√			
Function of daily life	Combing hairs	Have meals by himself	Washing perineum	Dress shoes
	5	5	5	10
	√	√	√	√
100	100			

## 3. Discussion

Coronal shear fractures of the distal humerus in teenagers remains to be a great challenge to orthopedic surgeons. The principles of fracture treatment in children are to relieve pain as soon as possible, anatomical reduction of the articular surface and rigid fixation of fractures to prevent late complications, and avoid deformity of the elbow joint due to epiphyseal injury.^[4]^

Shahram S Yari’s study showed that non-surgical or conservative management of coronal shear fractures of the distal humerus is ineffective and that such fractures should be treated surgically unless there is a contraindication to surgery.^[8]^ Michael Hackl and Arun Pal Singh found out that anatomical reduction of fracture is the key point for the recovery of elbow joint under adequate exposure of the fracture.^[9,10]^ Conventional surgical approaches for the treatment of such fractures entails wide exposure, which is not beneficial to the postoperative rehabilitation for the teenagers.^[11]^

The following 3 surgical approaches are commonly used to treat B3 fractures of the distal humerus: Lateral or posterolateral approach; Posterior approach: Olecranon osteotomy approach, triceps brachii approach; Anterior approach. The anterior approach is suitable for fractures involving the capitulum of the humerus and the involved articular surface of at the anterior aspect of trochlea.^[2]^ It can completely expose the fracture block, and an operating space large enough for the reduction and fixation of anti-slide plate, The surrounding soft tissues and blood supply could be satisfactorily protected despite of the process of reduction and fixation. The median nerve and brachial artery were protected carefully.

In addition, how to fix such fractures used to be 1 of the most difficult problems to be solved. Conventional treatments mostly adopted cannulated screws via joint surfaces,^[1]^ which were short and fixed in cancellous bone. With poor stability and difficulty in resisting the shear stress generated by the joint.,^[3]^ the cannulated screw would probably result in internal fixation failure and delayed healing, and even nonunion with necrosis of the distal humeral articular surface and elbow joint disability, and osteoarthrosis of the elbow.^[12]^

However, functional exercises within 3 weeks after surgical treatment is the key factor for functional recovery of the elbow joint.^[8,13]^ Studies have shown that the probability of elbow stiffness after trauma is at least 5%, and if the range of motion of the elbow is less than 30° to 120°, it will seriously affect orthobiosis.^[14]^ If functional exercise had been performed after complete healing of the fracturs, the limb is prone to suffer from apraxia osteoporosis, heterotopic ossification,^[15]^ muscle atrophy around the elbow joint,^[16]^ and adhesion of the elbow joint derived from hematoma. Therefore, the range of motion and function of the elbow will be remarkably restricted.^[13]^ More important, intra-articular fractures in children often involve epiphyseal injury.^[17]^ Repeated reduction and adjustment of internal fixation devices during surgery will further aggravate epiphyseal injury and disturb the growth of children’s The severity depend on the fracture end displacement and involvement of the growth plates which could result in malunion of fracture. Because of the low incidence of distal humeral coronal shear fractures and the age-related changes in the anatomical morphology of the distal humerus, the internal fixation devices for distal humerus are not readily available for children.^[4,18,19]^

The application of 3D printing designed customized plates can now be a perfect solution to the current problem of lacking appropriate internal fixator.^[20]^ Preparative 3D CT scans were performed on both the affected limb and the healthy side. of the patient’s elbow joint to reconstruct an equal proportion scaled fracture model. Individualized 3- dimensional printing designed anatomical plates that better match the patient’s biomechanical request were developed on the model under the principle of not affecting the smoothness of the joint surface and to avoid restricting the mobility of the elbow joint with satisfactory mechanical stability.^[21]^

Then the internal fixation is evaluated in vitro. The difficulty of fracture reduction and plate positioning were rehearsed repeatedly preoperatively. Therefore, intraoperative trauma reduced, surgery time decreased, surgical risk reduced.^[22]^ Moreover, the 3D-printing designed customized plate reduces the probability of heterotopic ossification of the elbow joint postoperatively by avoiding the possibility of taking out the plate for adjustment several times when the traditional internal fixation is poorly attached ^[20,23]^ The 3D-printing design customized plate fits perfectly with the distal humeral cortex, showing a stronger performance of shear force resistance, which enables the child to perform functional exercises as early as possible postoperatively, to prevents stiffness of the elbow joint and has great advantages for children’s postoperative functional recovery.^[24]^

3D printing technology has greater advantages in preoperative planning and surgical guidance.^[20]^The difficulty of fracture reduction and fixation can be practiced repeatedly on the in vitro 3D printing model preoperatively.^[25]^ Prior studies have revealed that the application of 3D printing technology in the surgical treatment of fractures can significantly reduce the operation time and blood loss.^[23]^ 3D printing technology first appeared in the 1970s, and Charles (Chuck) Hull is considered a pioneer of this technology.^[26]^ With the development of the design and manufacture technique, 3D printing designed customized plate will become increasingly mature, and will be an effective alternative at the special anatomical areas where satisfactory fracture fixation cannot be achieved by conventional commercial fixators.

The treatment of coronal shear fractures of the distal humerus in teenager is controversial at present. This report 3D printing technology designed customized plate in treatment of such fractures showed satisfactory results, which provides a feasible method for the treatment of fractures without suitable internal fixation devices in the future.

## Author contributions

**Data curation:** Haiyang Xing.

**Investigation:** Faxin Cao, Zhipeng Du.

**Supervision:** Gang Wang.

**Validation:** Gang Wang.

**Writing – original draft:** Changpeng Cao.

**Writing – review & editing:** XiyaoWang, Gang Wang.
